# How does context influence performance of community health workers in low- and middle-income countries? Evidence from the literature

**DOI:** 10.1186/s12961-015-0001-3

**Published:** 2015-03-07

**Authors:** Maryse C Kok, Sumit S Kane, Olivia Tulloch, Hermen Ormel, Sally Theobald, Marjolein Dieleman, Miriam Taegtmeyer, Jacqueline EW Broerse, Korrie AM de Koning

**Affiliations:** Royal Tropical Institute, P.O. Box 95001, 1090 HA Amsterdam, The Netherlands; VU University Amsterdam, Athena Institute for Research on Innovation and Communication in Health and Life Sciences, De Boelelaan, 1081 HV Amsterdam, The Netherlands; Department of International Public Health, Liverpool School of Tropical Medicine, Pembroke Place, Liverpool, L3 5QA UK

**Keywords:** Community health workers, Context, Equity, Gender, Health beliefs and practices, Low- and middle-income countries, Performance, Policy, Review

## Abstract

**Background:**

Community health workers (CHWs) are increasingly recognized as an integral component of the health workforce needed to achieve public health goals in low- and middle-income countries (LMICs). Many factors intersect to influence CHW performance. A systematic review with a narrative analysis was conducted to identify contextual factors influencing performance of CHWs.

**Methods:**

We searched six databases for quantitative, qualitative, and mixed-methods studies that included CHWs working in promotional, preventive or curative primary health care services in LMICs. We differentiated CHW performance outcome measures at two levels: CHW level and end-user level. Ninety-four studies met the inclusion criteria and were double read to extract data relevant to the context of CHW programmes. Thematic coding was conducted and evidence on five main categories of contextual factors influencing CHW performance was synthesized.

**Results:**

Few studies had the influence of contextual factors on CHW performance as their primary research focus. Contextual factors related to community (most prominently), economy, environment, and health system policy and practice were found to influence CHW performance. Socio-cultural factors (including gender norms and values and disease related stigma), safety and security and education and knowledge level of the target group were community factors that influenced CHW performance. Existence of a CHW policy, human resource policy legislation related to CHWs and political commitment were found to be influencing factors within the health system policy context. Health system practice factors included health service functionality, human resources provisions, level of decision-making, costs of health services, and the governance and coordination structure. All contextual factors can interact to shape CHW performance and affect the performance of CHW interventions or programmes.

**Conclusions:**

Research on CHW programmes often does not capture or explicitly discuss the context in which CHW interventions take place. This synthesis situates and discusses the influence of context on CHW and programme performance. Future health policy and systems research should better address the complexity of contextual influences on programmes. This insight can help policy makers and programme managers to develop CHW interventions that adequately address and respond to context to optimise performance.

**Electronic supplementary material:**

The online version of this article (doi:10.1186/s12961-015-0001-3) contains supplementary material, which is available to authorized users.

## Background

Community health workers (CHWs) are involved in the delivery of health services to the community and constitute the first point of contact on health-related issues in many low- and middle-income countries (LMICs). There are a wide variety of CHWs, with different names, working voluntarily or paid, with multiple or single and community-based or (partly) facility-based tasks [1,2]. CHWs have been defined as follows: “*Any health workers carrying out functions related to health care delivery; trained in some way in the context of the intervention, and having no formal professional or paraprofessional certificate or degree in tertiary education*” [3]. In addition, it is argued that CHWs “*should be members of the communities where they work, should be selected by the communities, should be answerable to the communities for their activities, should be supported by the health system but not necessarily a part of its organisation and have shorter training than professional workers*” [4].

Shortages in human resources for health and evidence that CHWs can significantly contribute to the health of the population by effectively delivering key interventions in primary and community health care have led to a renewed interest in CHW programmes in LMICs [1,3,5,6]. It is important to better understand the factors influencing performance of CHWs, since these are related to the success or failure of CHW programmes. Evidence on factors influencing CHW performance can help to improve CHW programme design and management.

Factors influencing CHW performance can be divided into intervention design factors that can be directly shaped and adjusted (such as supervision, incentives, training, and monitoring and evaluation mechanisms) and factors that represent the context in which a CHW intervention is taking place, which are less easily adjustable [5]. Research seldom focuses on the implications of context for CHW or programme performance [7]. Understanding the socio-cultural, economic, and political context in which CHW interventions operate is an important precondition for the design of successful interventions [2,5,8,9]. The health system in which CHW interventions take place often presents preconditions or limitations to the functionality of CHW programmes [1,5,9-11].

We conducted a systematic review with a narrative analysis on contextual factors influencing performance of CHWs, to contribute to the evidence-base on how these influence CHW or CHW programme performance. We make recommendations on the inclusion of context as an important element in CHW programme design and future research.

## Methodology

The literature review was part of a larger review that focused on both intervention design factors and contextual factors influencing the performance of close-to-community providers (presenting a wider range of health workers than CHWs, including auxiliary staff). For the purpose of this article, we focus on CHWs, as most of the evidence on contextual factors influencing performance was related to CHWs. We included quantitative, qualitative, and mixed-methods studies about CHWs working in promotional, preventive, or curative primary health care in LMICs. The studies should have described at least one factor related to the context in which CHWs work. The review covered studies including CHWs, their clients and their families/carers, CHW supervisors, the wider community, policy makers, program managers, other (professional) health workers, and any others directly involved in or affected by CHW service provision. We differentiated CHW performance outcome measures at two levels: CHW level (this included self-esteem, motivation, attitudes, competencies, guideline adherence, job satisfaction, and capacity to facilitate community agency as characteristics of performance) and end-user level (this included utilization of services, health-seeking behaviour, adoption of practices promoting health, and community empowerment as characteristics of performance) [10,12]. CHW level outcomes contribute to end-user level outcomes and both outcome levels constitute CHW performance, ultimately contributing to changes in the health of the population (impact) [13,14].

We searched EMBASE, PubMed, Cochrane, CINAHL, POPLINE, and NHS-EED for eligible studies. The search strategy was adapted from Lewin et al. [3] and is published elsewhere [13]. We searched reference lists of all relevant papers and reviews identified. We included English language studies from 2007 to July 2013, as the number of hits was large. We used a framework approach [15] and our preliminary conceptual framework [13] included predefined categories of contextual factors influencing CHW performance. These categories were: community context, policy context, health system factors, and other contextual factors and were based on reading of selected international literature [1,5,9,10,12,13,16-18]. A related review on intervention design factors influencing CHW performance is published elsewhere [13].

Two reviewers independently assessed the titles and abstracts of the identified records to evaluate their potential eligibility. In the case of different opinions, inclusion was discussed between the two reviewers until consensus was reached. The full-text papers were double assessed by a team of four reviewers.

We used a standardized data extraction form containing the description of the intervention, study, outcome measures, and the predefined contextual factors. The quality of included literature was assessed independently by two reviewers, with an adapted version of the Critical Appraisal Skills Programme method [19]. The quality assessment of studies was conducted to decide upon inclusion, but the level of quality was not taken into account during data analysis as the methodologies of included studies varied. Two reviewers analysed the content of included papers using thematic coding and the main categories of contextual factors influencing CHW performance from the preliminary conceptual framework were adjusted according to the findings [15].

## Results

### Results of the search

**Figure 1:**
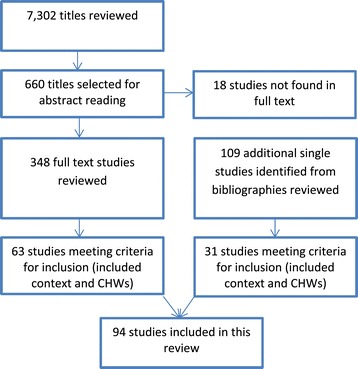
**Flow chart search results.**

The flow chart in Figure 1 presents the search results. The list of 94 included papers and their basic characteristics can be found in Additional file 1. A total of 42 studies were qualitative, 28 studies used mixed methods, and 24 studies were quantitative. Fifty of the studies were conducted in Africa and 41 in Asia. One study was from Oceania and two included Latin America. Most of the studies and interventions took place in rural settings. The programmes were run by either non-governmental organizations (NGOs) or governments or a collaboration of these. In 66 of the studies, the CHWs delivered services to people in their homes and/or in the community (as opposed to facility-based CHW services). CHWs in the included studies had diverse promotional, preventive, and curative tasks. Seventy-two studies reported outcomes at the level of the CHW and 35 studies at the level of the end-user. Few studies had the influence of contextual factors on CHW performance as primary research focus. However, many discussed these factors and we categorized them as factors related to the community, economic context, environment, health system policy, and health system practice.

### Community context

Community contextual factors that were found in the literature related to socio-cultural factors (including social and cultural norms, values, practices and beliefs, gender roles and norms, and disease-related stigma); safety and security and education and knowledge level of the target group (Table 1).

**Table 1:** **Summary of studies addressing contextual factors and their influence on CHW performance**

**Category**	**Sub-category**	**Sub-category**	**Detail on influence or association**	**Studies**
Community context	Socio-cultural factors	Social and cultural norms, values, practices and beliefs	Influencing health-seeking behaviour and therefore directly influencing utilization of CHW services or the ability of CHWs to reach the client	[20-32]
			Positively or negatively influencing acceptance of the CHW or the CHW intervention	[33-37]
			Not corresponding with CHW’s advice and therefore hindering CHW performance	[27,28,38-47]
			Social class of CHW could influence relationship between CHW and client	[48,49]
			Influencing level of initiative of the CHW	[33,50]
		Gender roles and norms	Influencing women’s access to and uptake of CHW services	[27,33,35,40,51-55]
			Preference regarding sex of the CHW influencing acceptance of the CHW	[32,40,56,57]
			Influencing possibilities for interactions of female CHW with male clients and therefore hampering CHW performance	[38,48,58]
			Influencing mobility of female CHW and therefore hampering CHW performance	[59,60]
			Influencing choice of becoming or retaining as a CHW (for example, women seen as caring, men should be paid, women discouraged to become CHW by husband)	[21,38,51,61-67]
		Disease related stigma	Influencing information provision to the CHW and health-seeking behaviour and therefore hampering CHW performance	[33,38,54,68-71]
	Safety and security		Feeling of unsafety could lower CHW motivation and conflict could hamper the functionality of programmes	[45,72,73]
	Education and knowledge level target group		Low education and knowledge levels of clients could hinder CHW performance	[33,57,74,75]
Economic context			Economic hardship could influence willingness to become CHW, health-seeking behaviour, and could lead to stress of CHWs	[55,62,64,74,76-78]
Environment	Geography and distance		Difficult geography and large distances to cover could hamper CHW performance	[20,24,25,38,45,55,72,79-82]
	Climate		Flooding could hamper mobility and thus performance of CHWs	[21,83-85]
Health system policy	CHW and human resources policy	CHW policy	Existence of CHW policy could influence CHW performance	[26,51,56,58,61,68,70,72,78,80,86-96]
		Human resources policy	Human resources policies, relating to incentives and career perspectives, influence CHW performance	[59,61,62,68,78,80,97,98]
	Legislation related to CHWs		Regulatory frameworks about procedures CHWs are authorized to perform could influence their scope of work and could influence their acceptability	[20,35,37,40,53,61,72,77,79,97,99-104]
	Political commitment		Political commitment towards CHW programmes could influence performance of CHWs	[58,72,81,92,105]
Health system practice	Health service functionality		Embedment of CHW services with functional, well-supplied health services could enhance CHW performance	[70,82,97]
			Functioning, bidirectional referral, and feedback systems enhance CHW performance	[71,106,107]
	Human resources provisions and their match with CHWs’ expectations		Expectations regarding career progression and incentives that are not corresponding with possibilities within health system could hinder CHW performance	[49,62,108-111]
			Well defined roles of CHWs and other workers could increase CHW performance	[80,97,110]
			Inadequate support of other staff or supervision could hinder CHW performance	[55,58,74,77,88,112,113]
			Outcome-based payment of CHWs could hinder their performance	[29,49]
	Level of decision-making		Decentralization could have an effect on performance of CHWs	[114]
	Costs of health services		User fees and income based on drug selling by CHWs could hinder their performance	[65,95,96]
	Governance/coordination structure		Hierarchical structures and too many vertical programmes could hinder CHW performance	[29,81]

#### Socio-cultural factors

##### Social and cultural norms, values, practices, and beliefs

We identified social and cultural norms, values, practices, and beliefs as important community contextual factors that affect CHW performance; these were particularly reported in studies related to maternal health programmes. For instance, women’s preference for giving birth at home was reported to be a deeply embedded cultural belief in Ethiopia, resulting in women choosing to deliver with a traditional birth attendant at home instead of with a health extension worker at a health post [26]. Similarly, lady health workers in Pakistan had difficulties in following-up newborns because of women delivering in their parents’ house and residing with them for 40 days after childbirth [23]. Likewise, seclusion of mother and baby after delivery was reported to hamper CHW performance in Bangladesh [21,24]. In many societies, the husband and mother-in-law are the primary decision-makers [25]. In India, grandmothers and mothers-in-law had a big influence on the health-seeking behaviour of pregnant women, often resulting in home births [28,29]. Two different studies on maternal health in Afghanistan and Bangladesh showed that involving the husbands [34,36], mothers-in-law [34,36], sisters-in-law, and mothers [36] in health education activities reinforced the messages of CHWs and enhanced coverage and acceptability of misoprostol.

Cultural practices, such as preference for herbal treatment, also influenced compliance with CHW guidance [45]. However, such practices did not always originate from a preference for traditional treatments but from modern treatments being unavailable [105].

Social hierarchies can also form a barrier to CHW performance. From India, Abbott et al. reported that female community based distributors (CBDs) faced challenges in influencing behaviour of women with a lower social status [48]. While in another setting in India, accredited social health activists (ASHAs) were in demand by all castes and religious groups [49]. Prata et al. reported that the social structures in Nigeria were extremely hierarchical and local leaders had strong influence on the “acceptability” of CHWs [35]. This, however, did not necessarily translate into constraints for the CHWs, there was still adequate community participation, and CHWs were still able to do their tasks (education about and distribution of misoprostol).

In Uganda, too, cultural and religious beliefs amongst the target groups made it difficult to approach them and this negatively influenced the level of initiative taken by community reproductive health workers [33]. CHWs’ initiative can also be positively influenced by social and cultural values. Community volunteer workers in palliative care in Uganda reported that the cultural desirability of and value attached to the act of helping each other underpinned their caring role for sick community members [50].

##### Gender roles and norms

Gender roles and norms, which intersect with social and cultural norms, influenced women’s access to and uptake of CHW services and thereby CHW programme performance. For example, in Swaziland, limitations on women’s agency and decision-making formed a barrier in access to HIV prevention and care interventions by CHWs [54]. A CHW intervention in Malawi on prevention of mother-to-child transmission of HIV found that women without any partner involvement were most likely to complete treatment. Those women with involved but undisclosed partners were least likely to complete treatment [52].

The sex of the CHW has been shown to influence uptake of services in different contexts. In Afghanistan, Viswanathan et al. reported a preference for female CHWs for the delivery of reproductive health services compared to male CHWs, because the norm was that women should not interact with men outside the family [56]. Hill et al. suggested that having only male community based surveillance volunteers (CBSVs) working in maternal and neonatal health in Ghana might have limited the scope of the intervention, as families may not want the CBSVs to physically help putting babies in the skin to skin position or help with breastfeeding attachment [40]. A family planning programme in Guinea recruited a female and male CBD per village. Only the female CBD, according to social custom, was allowed to approach women about family planning. However, male CBDs were able to engage with men and persuade them that family planning was also a men’s concern [57]. In India, female CBDs working in promotion and distribution of contraceptives were limited in their interaction with men, which hampered their performance. This was a result of the norms of *purdah*, which strictly regulates interaction between men and women [48]. The same was found for women health volunteers in Iran [58]. Being female could influence mobility of CHWs: two studies from Bangladesh reported that *Shasthya Shebikas* (CHWs) were seen as being “not decent” if they went out in the night [59,60], particularly in rural areas [59].

Gender norms and roles affect expectations for income generation of men and women and can influence people to become or remain a CHW. In patriarchal settings, men are expected to be the family breadwinners. A study in Kenya, for example, showed that for this reason, it became difficult for male CHWs to provide voluntary services as it strained their ability to fulfil their financial responsibilities. As a result, they were forced to drop out to search for alternative sources of income. This cultural norm was not the only reason for the higher drop out of male CHWs as compared to female CHWs; it was also indicated that men lacked certain characteristics like instinct for tender care and tolerance that a sick person requires, whereas female CHWs believed “it is their natural duty” to care. Although remuneration was not as strong a condition for women as for men to become a CHW, lack of remuneration and sometimes lack of spousal support (women were perceived to “waste time” if they engaged in community work) were reasons to drop out for female CHWs [63]. Results of a survey conducted with 764 female *Shasthya Shebikas* in Bangladesh showed that 5.9% faced problems in obtaining permission from their husbands for participation in this mainly voluntary job [65].

##### Disease-related stigma

Several studies reported disease related stigma influencing the performance of CHWs. In a project involving peer counsellors to support clients to adhere to anti-retroviral therapy (ART) in Ethiopia and Uganda, peer counsellors’ performance was limited by some clients not disclosing contact details through fear of having their HIV status known [70]. Stigma also played a role in Uganda, where CHWs found it difficult to approach clients about family planning [33], and in Kenya, where trained HIV infected peers delivering HIV care at household level defined themselves as health counsellors to avoid the AIDS label and promote confidentiality [71].

#### Safety and security

In addition to constraints for female CHWs as mentioned above, safety and security issues may also affect their performance. A study in Papua New Guinea, describing the social factors that influence motivation of rural health workers, addressed work safety issues as a factor influencing CHW performance. A perceived lack of personal safety was found to affect motivation to work at particular locations and, in some cases resulted in people resigning. Especially (young) female health workers felt unsafe and scared, because of substance abuse among young men, violent assaults, verbal abuse, and accusations [45]. Callaghan-Koru et al. reported that health surveillance assistants (HSAs) running village clinics in Malawi were sometimes afraid of contracting infections and the possibility of stealing of drugs by community members [72]. Teela et al. reported that security concerns (because of an active conflict) could substantially impinge on the service provision of maternal health workers in Myanmar. The authors reported that the flexible nature of the multi-tiered provider network was able to partially overcome security constraints and maintain coverage of some services [73].

#### Education and knowledge level of the target group

Low levels of education and health knowledge in the population were reported to pose a challenge for CHWs in Kenya, who perceived some people in their communities to be “ignorant” and “uncooperative” [74]. Community reproductive health workers in Uganda reported that misconceptions about contraception were the major factors hindering their work [33]. However, this could be interpreted as an attitude of the CHW rather than a contextual factor, which will be further elaborated in the discussion section.

### Economic context

The economic context and its influence on the performance of CHWs were highlighted in a number of studies; they related mainly to livelihoods and willingness to volunteer, and requested compensation for services rendered (Table 1). A lack of financial or material compensation for services rendered could lead to an inability of CHWs to provide for their family and is particularly exacerbated in areas of pervasive poverty [62]. The willingness to become a CHW could be influenced by the wish to earn an income or the hope of being compensated eventually, especially in situations where there is high unemployment or fewer opportunities [64,76-78]. For example, a study on CBDs in Uganda reported that, due to high levels of unemployment, people volunteered hoping that they would be remunerated eventually [77]. Poverty of the community could also influence the work of CHWs. Maes et al. reported that a food crisis not only affected CHWs, but also led to lack of food among clients causing distress to CHWs (because they saw their clients suffering) [62]. Poverty could also prevent people from seeking health services in general, because of the expense incurred for accessing the services [55].

### Environment

Several studies reported that topographical challenges and the need to cover large distances hampered CHW performance. Mukanga et al., in a study on CHWs working in child health in Uganda, found that households residing 1 to 3 km from a health facility were 72% more likely to utilize CHW services compared to households residing within 1 km of a health facility [79]. Households residing between 1 and 3 km from a CHW were 81% less likely to utilize CHW services compared to those households residing within 1 km of a CHW. Thus, proximity of CHWs and health facilities to their clients could affect utilization of CHW services [79]. Four studies referred to difficulties of CHWs in reaching communities because of flooding, which hampered their performance [21,83-85] (Table 1).

### Health system policy

The literature revealed four key contextual factors relating to health system policy having a bearing on CHW performance: the existence of a CHW policy, a human resources policy, legislation related to CHWs, and political commitment (Table 1).

#### Existence of CHW and human resources policies

Authors mentioned the importance of having a national CHW policy in studies from several countries: Pakistan [51,89,92,93], Afghanistan [36,56], Malawi [72,96], India [86], Ethiopia [26,80,87], Iran [58], and South Africa [78]. In Thailand and Bolivia, there was no clear policy for community health care workers and *manzaneras*, respectively [88,94]. This lack of policy led to inadequate support for CHWs (credits and payments for trainings) [94] and to CHWs not being recognized by health authorities, which limited their ability to operate in the community [88]. In South Africa, although a national CHW policy framework was adopted, most CHWs are not employed by the government and challenges regarding support to and management of CHWs still exist [78]. Furth et al. reported that, in Zambia, the government recognizes CHWs, but the health system is still not equipped to supervise, support, and incentivize the full range of CHWs operating in the country [90].

General human resource policies define the space in which programmes and interventions can operate regarding incentives, working conditions, training, and career perspectives. Therefore, human resource policies can have an effect on CHW performance. The literature review found that, in many contexts, the rights of CHWs were not formally covered. There was a lack of basic entitlements such as leave and complaint mechanisms for CHWs [78]. Policies addressing remuneration and incentives were lacking in some contexts [62]. In Uganda, the lack of a regulatory framework resulted in fragmentation of salaries among different types of CHWs and lack of career opportunities, resulting in demotivation [68,70]. In Ethiopia [62,80] and Mozambique [62], a clear professional development programme was reported to be absent.

#### Existence of legislation related to CHWs

The medical profession is regulated and restricted in all countries; legislative and professional regulatory frameworks inform which professional can perform which task. Few studies however reported on regulatory frameworks regarding the health-related procedures CHWs are authorized to perform. Callaghan-Koru et al. presented an example of disagreement at national level in Malawi about CHW services, when in 2009, the Medical Council considered the community case management programme to be illegal because they had objections to HSAs performing clinical services [72]. The work of CHWs in Bangladesh was facilitated by the fact that they were permitted to prescribe medication [53]. In Zambia, the policy on HIV counselling and testing services changed so that lay counsellors could test clients [102], and Nepal was reported to change the policy in order to make it possible for CHWs to prescribe antibiotics [104]. Nigeria became the first country in the world to approve national guidelines for the prevention and treatment of post-partum haemorrhage allowing community-based distribution of misoprostol [35]. Thus, regulatory frameworks guide the scope of the activities that CHWs are allowed to perform.

#### Political commitment

On occasion general political decisions can influence CHW performance. In India, the influence of local politics in selecting local people to manage community-based drug distribution centres caused deterioration of the centres and negatively influenced the ability of CHWs to conduct their job [105]. Regarding a CHW programme in Iran, leadership and continuous support of the formal health system were central to the success of the intervention [58].

### Health system practice

There were several factors affecting CHW or programme performance related to the health system practice: health service functionality (including supplies), human resources provisions, the level of decision-making, the costs of health services, and the governance and coordination structure (Table 1).

#### Health service functionality

Many studies stated that the presence of well-functioning health services is essential for CHWs to perform well, including logistics support, equipment, and supplies. For example, peer counsellors in Ethiopia had sincere concern for their fellow patients, which resulted in frustration when they observed sick patients not being initiated on ART due to lack of drugs, and how tired and hungry patients waited for long hours to be counselled by a provider [70]. Drawing on research in Uganda and Lesotho, Dambisya et al. [97] and Satti et al. [82] highlighted the importance of embedding CHW service delivery within a continuum of services. A functioning and bidirectional referral and feedback loop was also mentioned to enhance CHW performance [71,106,107].

#### Human resources provisions and their match with CHWs’ expectations

Studies showed that CHW motivation could be influenced by the health system’s ability to accommodate CHWs’ expectations – particularly around formalization of their status, prospects of career development, and incentives. The literature revealed that CHWs find monetary and material incentives important. The perspective of working towards a permanent job is, for many, an important incentive as well, while in some settings CHWs have become a formal cadre [49,108,111]. Peltzer et al. reported that voluntary lay counsellors in South Africa are unlikely to continue to serve without salaries – particularly if the range of tasks expected from them is broadened [110]. Unkept promises regarding future incentives may dissatisfy CHWs (although not necessarily lead to attrition) [62], and parallel programmes offering incentives when their own does not, may discourage them [109].

Studies showed that for CHWs to be effective, they should have clear operating procedures and guidelines, including clearly defined and demarcated roles and relationships with other cadres and actors [80,97,110]. The absence of support from professional health staff, e.g., due to high professional staff turnover [112], lack of trust by professional staff [55,88], overall lack of such support [74], a general lack of professional staff [58], and workload of other staff [77] can affect motivation and performance of CHWs. Lack of support from or supervision by the health system can also result in lack of credibility of CHWs [88]. Scott et al. reported the negative side of outcome-based payment of ASHAs in India [29]. ASHAs earn money for bringing people to the clinic and helping with biomedical interventions. They cannot earn money for encouraging village health meetings nor discussing health issues on social change more generally, while this is part of their role. This resulted in underperformance on these particular tasks [29,49].

#### Level of decision-making

The level at which decision-making occurs and the implementation capacity at those levels could have an influence on CHW performance. In Laos, responsibility for public programmes was shifted from the central level to provincial and district levels. Although this shift did, in principle, recognize the value of community engagement and locally designed interventions, low capacity at these levels meant that programmes were often poorly managed, non-consultative, and not evidence-based, and that activities were regularly carried out in an *ad hoc* fashion. There was also a major dependency on donor funding and expatriate expertise. These problems as a result of a shift in responsibility could affect performance of CHWs [114].

#### Costs of health services

The costs of health services could also have an effect on CHW performance. In Malawi, a study reported that HSAs in a catchment area managed by a faith-based association were unable to collect drugs from their nearest facility as they were not free of charge like in government catchment areas. Therefore, their performance regarding community case management of childhood illness was constrained [96]. CHWs in Mali, who obtained income by selling drugs, had to compete with informal vendors that sold drugs in smaller, cheaper quantities [95]. *Shasthya Shebikas* in Bangladesh also faced competition regarding selling medicines [65]. As a result, community use of CHW services was low in these settings.

#### Governance and coordination structure

Few studies discussed the governance and coordination structure influencing CHW performance. A hierarchical structure of the health system hindered meaningful communication across levels of status, seniority, and income in India. This rigidity and top-down power and information flow had negative effects on the ASHA programme, as insights from the community level were not used to improve services at all levels [29]. In Ethiopia, lack of coordination between vertical programmes and between various NGOs was reported to result in overlap among different (*ad hoc*) trainings, reducing the time health extension workers could spend in their communities [81].

## Discussion

**Figure 2:**
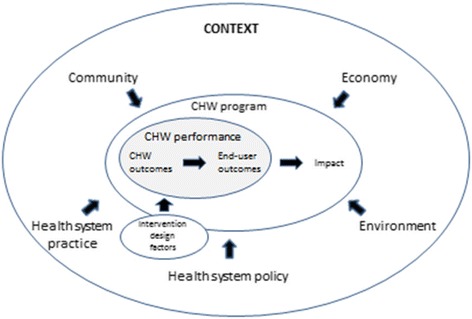
**Contextual factors influencing CHW performance and programme functionality.**

Our findings indicate that contextual factors influence CHW performance at the CHW level (e.g., motivation or competencies), the end-user level (e.g., influencing health-seeking behaviour), or by influencing broader CHW programme performance (Figure 2). These factors relate to community (most prominently), economy, environment, and health system policy and practice and form a complex interactive web. They represent characteristics of settings in which a CHW programme operates and sometimes serve as preconditions for the performance of CHWs or CHW programmes. Factors that were found to be preconditions, such as the presence of well-functioning health services including logistics and supplies, were affecting CHWs’ ability to conduct their job. They were also related to CHW and end-user outcome levels, selected as outcome measures and comprising CHW performance in this review. For example, the absence of well-functioning health services influenced levels of motivation and job satisfaction (at the CHW outcome level) and utilization of services at the end-user outcome level. Policy makers and implementers of CHW interventions have to anticipate on and make use of the context particular to their setting to reach optimal performance.

Socio-cultural factors can influence access to and uptake of (CHW) health services. As CHWs are part of the context in which clients are living, it is often assumed that they are better able to understand constraints as a result of socio-cultural factors, compared with other health workers [1,5]. Findings from our review are similar to those of a systematic review of community participation in malaria control [115]; this review showed that, in certain cases, CHWs were able to shift health-seeking behaviour towards increased utilization of services, but in others they were not. Contextual analyses are needed to understand how CHWs could be better used to stimulate health care seeking and reduce stigma or modify socio-cultural norms and beliefs amongst different groups. At the same time, reflection on norms and values and addressing power relations are rarely the focus of CHW interventions, which could explain why it is difficult for CHWs to address these issues. Poor adherence to the CHW’s advice or poor health-seeking behaviour could be exacerbated by low education levels of the target group [116]. Lack of skills of the CHW to use effective health education strategies and community dialogue interventions may lead to a lack of compliance with their advice, frustration, and blaming the client to be ‘ignorant’. Understanding of community practices and beliefs could assist policy makers in shaping CHW programmes, for example, providing training and supervision to equip CHWs with the right facilitation skills to initiate reflection on practices and beliefs that, from a public health perspective, hinder people from changing behaviour or accessing services.

Our review identified the influence of social hierarchies on CHW acceptance and performance. Palazuelos et al. emphasized the importance of structures and dynamics of societal interaction; the way power is shared within a society and the trust people have in those with power as factors influencing performance of CHW programmes [9]. Recently, a South African study reported unintended CHW programme outcomes as a result of multi-layered practices of power [117]. In communities where government or local leaders have a big influence, it could be advisable to involve them in the CHW programme, although in some contexts this could perpetuate existing social inequities and power imbalances. Thus, there is a delicate balance between working with and influencing context.

Social and cultural norms should be taken into consideration when selecting CHWs to address community preferences regarding sex and social status. Some communities prefer female CHWs yet they may be less able to perform because of societal and gendered restrictions in mobility or communication with male clients. Gender roles and relations shape processes and experiences within the community and within the health system and CHWs have a critical interface role between both sides. Mumtaz et al. reported that lady health workers in Pakistan were introduced because they were assumed to be better able to respond to gender-based constraints on women’s access to health services, but they themselves faced problems because they operated in the same gender systems that necessitated their appointment [118,119]. This led to low job satisfaction and significant negative implications for the quality of services provided. Gender norms could also influence decisions on becoming or retaining a CHW. This could be a consideration when designing voluntary CHW programmes, especially in patriarchal, poor societies where men are not easily involved in voluntary work. Involvement of husbands was found to enhance coverage of CHW services in maternal health [34,36]. Although many studies point to the beneficial effect of male involvement in programmes for the prevention of mother to child transmission of HIV [120], a study from Malawi showed low adherence to HIV treatment of women with husbands that did not know the HIV status of their wives [52]. This highlights the importance of understanding how gender roles and relations influence health-seeking behaviour and responding appropriately. CHWs thus need to be supported to be able to assess and react to this context in promoting health-seeking behaviour.

Stigma is a culturally specific construct that could influence CHW performance. When stigma is profound, certain CHW programmes should be adjusted to improve performance, for example by integration of HIV services into other health services to avoid patients being identified [121].

Existence of CHW-related policies was found to be important. However, recognition and integration of CHWs in the health system seem to be more important for CHW performance than the existence of a CHW policy *per se* [78,90]. The importance of inserting CHW programmes in the wider health system and the human resource strategic planning has also been identified by others [1,10,11]. Formally employed CHWs are more likely than voluntary CHWs to be covered by policies and frameworks regulating their rights, this promotes sustainability of programmes.

Regulatory frameworks regarding the procedures CHWs are allowed to perform will become more important when CHWs become involved in providing curative services [2]. These regulatory frameworks should protect CHWs in case of adverse outcomes.

The functionality of the health system as a whole has an influence on CHW performance. From our literature review, it is clear that necessary arrangements regarding incentives, supervision, referral, supplies, and training are often inadequate and that CHWs’ expectations regarding these issues do not correspond with reality [1,9-11,116,122,123]. Performance- or output-based incentives could lead to competition or neglect of unpaid tasks, hampering CHW performance [29,49]. Hierarchical structures and vertical programmes within the health system hamper communication among CHWs, other health staff and management, and among NGOs that employ CHWs, negatively affecting CHW performance. Certain characteristics of vertical programmes, such as clear objectives and work schedules and frequent supervision, are assumed to facilitate performance [124]. However the existence of multiple vertical programmes could also lead to confusion at the community level as a result of unclear division of roles and responsibilities of different types of CHWs involved in these programmes and to dissatisfaction at the CHW level, because of differences in policies regarding incentives and career advancement. Thus, multiple vertical programmes could negatively influence CHW performance.

A limitation of this literature review is that we may have omitted studies, because only English language studies from 2007 to 2013 were included. We did not identify many studies reporting on power structures, the history of community organizations or structures, the role of professional associations, political commitment, and accountability structures. The categorization of factors as presented in this paper is based on a health systems perspective and is not static. Broader factors such as non-health-related governance structures, policy, and justice issues, and societal perspectives on volunteerism were not found in the selected literature, but might be factors that could influence CHW or programme performance. The quality of included studies was assessed to decide on inclusion but not used to ‘weigh’ the synthesis towards the findings of included studies, because of the wide range of types of studies included. Another limitation of this review is that in many of the included studies, the context and specific characteristics of CHWs were poorly described. Often, studies lacked “thick descriptions” [125], making it difficult for the reader to assess the relevance and depth of detail of context as well as similarities and differences across and between various contexts. Therefore, it is difficult to generalize the findings from one study to other settings (inferential generalization) and to draw theoretical propositions, principles, or statements from the findings of a study for more general application (theoretical or analytical generalization) [126,127]. Despite this, we have been able to identify some socio-cultural-related factors that influence performance of CHWs across different settings, supporting a discussion of theoretical generalizability.

The importance of describing context has been endorsed before in CHW related research [3,7,12,116]. Health systems are part of their social, political, and economic settings, responding to health needs that are generated by the same contextual factors. Context influences the daily practice of the health system through the experiences, mind-sets, and values that shape the behaviour of actors within it and, therefore, despite similar elements and patterns, they can respond differently to the same new idea, policy, or intervention. In order to bring positive change to health systems, health policy and systems research that fully accounts for context is required [128]. With regard to research on CHW performance, CHWs’ interface role makes it important to understand context from within communities and the health system, which are again shaped by policy and other factors. Therefore, qualitative and theory-driven approaches in research and evaluation of CHW programmes are recommended. Research which includes adequate descriptions and analysis of context is needed to provide evidence on the influence of contextual factors on CHW performance as the current evidence is not sufficient to assist policy makers to develop CHW programmes and interventions that anticipate or make use of context.

## Conclusions

This systematic synthesis of evidence shows that contextual factors related to community (most prominently), economy, environment, and health system policy and practice can influence CHW performance. Contextual factors can interact with each other to shape CHW performance and affect the performance of CHW interventions or programmes. While the current body of research often does not capture and explain the context in which CHW interventions take place, this synthesis, given its wide scope, provides understanding of the influence of context on CHW and programme performance. Future health policy and systems research should better address the complexity and the influence of context to support policy makers and programme managers to improve CHW interventions.

## Additional file

Additional file 1:
**Included studies and their basic characteristics.** This additional file gives a detailed overview of the 94 included studies of the review.

## Abbreviations

ART: Anti-retroviral therapy
ASHA: Accredited social health activist
CBD: Community based distributor
CBSV: Community based surveillance volunteer
CHW: Community health worker
HSA: Health surveillance assistant
LMIC: Low- and middle-income country
NGO: Non-governmental organization
