# Supplementary material for: How does context influence performance of community health workers in low- and middle-income countries? Evidence from the literature
Source: Health Res Policy Syst. 2015 Mar 7;13:13. doi: 10.1186/s12961-015-0001-3 (PMC4358881; doi:10.1186/s12961-015-0001-3)
Supplement: Additional file 1: — Included studies and their basic characteristics. This additional file gives a detailed overview of the 94 included studies of the review. [file 12961_2015_1_MOESM1_ESM.pdf]

Additional file 1. Included studies and their basic characteristics

First author, year and reference number	Country	CHW	Health focus	Study objective	Study type
Abbot 2011 [48]	India	Community Based Distributors (CBDs)	Reproductive health<br><br>Distributing contraceptives, referral	To uncover the conflicting expectations that many CBDs experience: to comply with project objectives without violating local social norms that limit interactions across status boundaries	Qualitative (participant observation)
Agrawal 2012 [86]	India	AWWs (Anganwadi workers)	Maternal health, family planning, child care<br><br>Preventive and curative	To explore the relationship between the knowledge level of CHWs (AWWs and ANMs) and their antenatal home visit coverage and effectiveness of the visits, in terms of essential newborn health care practices at the household level in rural India	Quantitative
Ahmed 2008 [67]	Bangladesh	*Shasthya Sebikas* (SSs)	Multiple preventive and basic curative services	To describe the story of the SSs - the BRAC model of sustainable community health workers, including its problems and prospects	Qualitative (descriptive)
Ajayi 2008 [20]	Nigeria	Community Medicine Distributors (CMDs)	Malaria in children less than 5 years<br><br>Drug distribution and explanation	To determine the feasibility of introducing a new antimalarial drug to the community, especially in rural areas, and assess the community perception on its effectiveness	Qualitative
Alam 2012c [59]	Bangladesh	CHWs	Maternal and neonatal health<br><br>Promotion, preventive	To assess factors related to retention of CHWs in urban slums in Dhaka where BRAC has implemented the Manoshi project	Mixed methods
Alamo 2012 [68]	Uganda	Community antiretroviral therapy and tuberculosis treatment supporters (CATTS)	HIV<br><br>Support for adherence, referral	To assess the performance of CATTS in scaling up ART in Reach Out, a community-based programme in Uganda	Mixed methods
Amare 2011 [87]	Ethiopia	Volunteer CHWs (vCHWs)	Multiple	To document the implementation of the introduction of several non-financial incentives for vCHWs and engagement of community anchors to support vCHWs and assess their effectiveness	Qualitative

Azad 2010 [21]	Bangladesh	Traditional Birth Attendants (TBAs) and facilitators of women groups	Maternal and neonatal health<br><br>Preventive Referral, support	To test the generalizability and scalability of a community-based participatory approach with women's groups to address maternal and neonatal care in three rural districts of Bangladesh	Quantitative
Baqui 2009c [22]	Bangladesh	CHWs	Neonatal health<br><br>Preventive, curative	To assess the effect of the timing of first postnatal home visit by community health workers on neonatal mortality	Quantitative
Bartos 2009 [88]	Bolivia	CHWs (called *manzaneras de la salud* in local language)	Maternal and neonatal health<br><br>Promotion	To evaluate a programme with the aim to extend the duration of breastfeeding in children less than 6 months living in the area of the Corea Municipal Health Network, El Alto, Bolivia	Mixed methods
Behdjat 2009 [58]	Iran	Women Health Volunteers (WHVs) (urban CHWs)	Promotion of using participatory approaches	To show the application of action research to inform policy-makers about potential changes in health care delivery and to describe and analyse a pilot project that refocuses on the tasks of urban CHWs in the Islamic Republic of Iran	Qualitative
Bhutta 2008 [89]	Pakistan	Lady Health Workers (LHWs), TBAs ('Dais')	Maternal, neonatal and child health<br><br>Promotion, prevention	To investigate the feasibility of delivering a package of community-based interventions for improving perinatal care using LHWs and TBAs (Dais) in rural Pakistan	Mixed methods
Bhutta 2011 [23]	Pakistan	Lady Health Workers (LHWs), voluntary Community Health Committees (CHCs) and TBAs ('Dais')	Perinatal and newborn care<br><br>Primary care, promotion, prevention	To evaluate the effectiveness of a community-based intervention package, principally delivered through LHWs working with TBAs and community health committees, for reduction of perinatal and neonatal mortality in a rural district of Pakistan	Quantitative (cluster randomized effectiveness trial)
Burn 2008 [51]	Pakistan	Lady Health Workers (LHWs)	Basic health services (with emphasis on women and child health)	To discover and explore factors that cause LHWs to resign from Pakistan's LHW Programme by gaining an insight into the experiences and opinions of resigned LHWs and understanding how these impacted on their decision to leave the programme	Qualitative

Callaghan-Koru 2012 [72]	Malawi	Health Surveillance Assistants (HSAs)	Childhood illnesses, family planning, tuberculosis, voluntary counselling and testing for HIV<br><br>Community case management	To explore health workers and managers perceptions about community case management provided by HSAs during the programme's first year in Malawi	Qualitative
Callaghan-Koru 2013 [96]	Malawi	Health Surveillance Assistants (HSAs)	Childhood illnesses<br><br>Case management of childhood illnesses	To assess selected health systems support (supervision, drug supply and job aids) for a national community case management programme for childhood illnesses in Malawi during the first year of implementation	Mixed methods
Campbell 2008 [38]	South Africa	Volunteers	HIV and AIDS<br><br>Promotion, home based care	To report on community perceptions of a 3-year project which sought to train and support volunteer health workers in a rural community in South Africa	Qualitative
Chanda 2011 [106]	Zambia	CHWs	Malaria<br><br>Promotion, prevention, treatment, referral in case of complicated malaria	To generate information on the capacity of CHWs to use rapid diagnostic tests and artemisinin-based combination therapy as effective tools for Home Management of Malaria (HMM). It was anticipated that results from this study could inform policy on the feasibility and effectiveness of a large scale HMM programmes implemented by CHWs.	Mixed methods (prospective evaluation)
Dambisya 2012 [97]	Uganda	CHWs	HIV, family planning, sexual and reproductive health<br><br>Prevention, counselling	To assess the policy and programmatic implications of task shifting in Uganda	Qualitative
Daniels 2012 [61]	South Africa	Lay Health Workers (LHWs)	Multiple<br><br>Prevention, curative	To explore the contemporary development of LHW policy in South Africa and to explain how gender was considered in this process	Qualitative
Darmstadt 2010b [24]	Bangladesh	CHWs	ANC, postnatal care, IMCI<br><br>Home visits, referral	To examine outcomes of the surveillance programme including 1) factors associated with coverage of postnatal assessment by CHWs and 2) factors associated with compliance with referral by CHWs	Quantitative

Das 2008 [105]	India	Village Volunteers, Anganwadi workers	Malaria<br><br>Promotion, preventive, curative	To assess the feasibility of establishing drug distribution centres through village volunteers in a tribal area in India where health-seeking practice of the community has been poor and to assess the impact of treatment of fever cases with chloroquine on morbidity, mortality and parasite prevalence in the community	Quantitative
Dawad 2011 [69]	South Africa	Community Rehabilitation Facilitators (CRFs)	Rehabilitation (care for people with disabilities)<br><br>Referral, awareness raising, care	To identify lessons to be learnt from Community Based Rehabilitation programmes using multi-skilled mid-level workers for increasing access to HIV care for people living in low-income rural areas without easily accessible health care infrastructure	Qualitative
Dawson 2008 [104]	Nepal	Female Community Health Volunteers (FCHVs)	Child health, pneumonia<br><br>Prevention, curative	To describe Nepal's efforts, starting from the mid-1980s, to develop and implement community-based management of pneumonia	Qualitative (descriptive)
Diakite 2009 [57]	Guinea	Community-based distributors (who are part of the Village Health Committees (VHCs)	Family planning<br><br>Promotion	To highlight the VHC in the child survival project and the integration of family planning work, describe the VHC purpose, membership, and tasks, and conclude with some outcomes of Save the Children's family planning component in Mandiana and Kouroussa districts, Congo	Qualitative (descriptive)
Elmardi 2009 [83]	Sudan	Malaria Control Assistants (MCAs)	Malaria<br><br>Diagnosis, treatment, home-based	To assess the feasibility and acceptability of home-based management of malaria (HMM) strategy using artemisinin-based combination therapy for treatment and rapid diagnostic test for diagnosis	Mixed methods
Furth 2012 [90]	Zambia	CHWs	HIV and AIDS<br><br>Promotion, curative, counselling (positive living and anti-retroviral treatment adherence counselling)	To test the hypothesis that by applying the CHW AIM tool and addressing programme weaknesses identified through the process, organizations will be able to improve the functionality of their CHW programmes. 3 Key questions formed the foundation for the CHW AIM operations research activity: 1 Does application of the CHW AIM tool contribute to CHW programme functionality improvement; 2 what is the relationship between programme functionality, CHW engagement and CHW performance; 3 what are the costs associated with implementing the CHW AIM tool and what is the incremental cost effectiveness associated with its use?	Mixed methods

Ge 2011 [98]	China	CHWs	Multiple	To clarify the level of job satisfaction of Chinese CHWs between a metropolitan (Shenyang) and a small city (Benxi) in Liaoning province, China and explore its associated factors	Quantitative
Gill 2012 [39]	Zambia	Traditional Birth Attendants (TBAs)	Neonatal health<br><br>Prevention, promotion, curative	To provide relevant details on how interventions in the Lufwanyama Neonatal Survival Project were developed and how Zambian TBAs were trained to perform them	Qualitative
Gusdal 2011 [70]	Ethiopia, Uganda	Peer counsellors	HIV<br><br>Support, anti-retroviral treatment adherence	To explore peer counsellors' work and their role in supporting patients' adherence to anti-retroviral treatment in resource-limited settings in Ethiopia and Uganda	Qualitative
Hill 2008 [40]	Ghana	Community Based Surveillance Volunteers (CBSVs)	Neonatal health<br><br>Promotion, curative	To provide information on intervention design by describing the process and information used to design a large scale community-based newborn intervention in Ghana (called Newhints)	Mixed methods
Hoke 2008 [99]	Madagascar	Community Based Distribution (CBD) workers	Family planning using injectable contraceptives<br><br>Prevention, distribution	To inform the MOHFPSP (Ministry of Health, Family Planning and Social Protection) officials with evidence that CBD of depot-medroxyprogesterone acetate could be provided safely and effectively by lay health workers in Madagascar	Mixed methods
Hoy 2008 [114]	Laos	Village youth volunteers	HIV and AIDS, sexual transmittable infections<br><br>Peer education, promotion	To assess the outcomes of the Lao Youth HIV and STI Response Project at the district and village levels in terms of: (1) the capacity of district project working teams to develop and implement their HIV and STI activity plans; (2) the sustainability of the project; and (3) the ability of young Lao people to respond to the risk of HIV and STIs through healthy behaviour change	Qualitative
Huber 2010 [100]	Afghanistan	CHWs	Family planning<br><br>Promotion, contraceptive injections	To assess the increase in contraceptive use in rural Afghanistan	Mixed methods
Jack 2012 [50]	Uganda	Community Volunteer Workers	Palliative care	To evaluate the motivation for becoming a volunteer and the personal impact of being a palliative care Community Volunteer Worker in Uganda	Qualitative

Javanparast 2011b [108]	Iran	CHWs	Multiple	To explore the perceptions of CHWs regarding their contribution to rural health in Iran	Qualitative
Javanparast 2012 [91]	Iran	CHWs	Female CHWs assist deliveries<br><br>Prevention, promotion	To analyse the CHW training process in Iran and how different components of training have impacted on CHW performance and satisfaction	Qualitative
Kalyango 2012 [101]	Uganda	Community Medicine Distributors (CMDs)	Integrated case management of childhood illnesses of Malaria and pneumonia<br><br>Curative	To compare the performance of CHWs in the dual management of malaria and pneumonia versus CHW management of malaria alone in children under five and to assess the factors influencing CHW performance	Mixed methods
Kim 2012b [52]	Malawi	CHWs	HIV<br><br>Care regarding prevention of mother to child transmission (PMTCT)	To evaluate a CHW based patient case management system for PMTCT	Quantitative (cohort study)
Kok 2013 [75]	Malawi	Health Surveillance Assistants (HSAs)	Multiple	To assess motivation, job perception and satisfaction of HSAs in Mwanza district, Malawi	Qualitative
Maes 2013 [62]	Ethiopia, Mozambique	CHWs	HIV and AIDS<br><br>Prevention, promotion, recruitment of patients, support	To provide policymakers with a holistic understanding of how and why people become and remain CHWs and to generate in depth understanding of life histories that lead people to become CHWs, their reasons to stay CHWs in particular their relationships with intended beneficiaries after becoming CHWs and their social and economic aspirations	Qualitative
Mannan 2008 [41]	Bangladesh	CHWs	Maternal and neonatal health<br><br>Promotion	To study whether postpartum visits by trained CHWs reduce newborn breastfeeding problems	Quantitative (cluster randomized controlled trial)
Martinez 2008 [33]	Uganda	Community Reproductive Health Workers (CRHWs)	Reproductive health<br><br>Promotion	To assess the current practices, barriers and training needs of CRHWs	Qualitative

McPherson 2010 [25]	Nepal	Female Community Health Volunteers (FCHVs)	Maternal and neonatal health<br><br>Health promotion	To assess the assumption that FCHVs are effective at promoting care-seeking and desired household practices and that a booklet will facilitate the communication of messages, reinforce the FCHVs' counselling and stimulate intra-household discussion	Qualitative
Medhanyie 2012 [26]	Ethiopia	Health Extension Workers (HEWs)	Multiple health focus	To assess utilization to maternal health services by women in rural villages in Ethiopia	Quantitative (cross sectional survey)
Mukanga 2010 [42]	Uganda	Community Medicine Distributors (CMDs)	Malaria<br><br>Curative	To assess community acceptability of the use of rapid diagnostic tests by CMDs in Uganda	Qualitative
Mukanga 2012 [79]	Uganda	CHWs	Malaria, pneumonia<br><br>Community case management (for children under 5)	To assess household access, utilization and acceptability of the use of Rapid Diagnostic Tests (RDTs) and Respiratory Rate Timers by CHWs following one year of implementation	Quantitative (cross sectional household survey)
Mukherjee 2007 [76]	Focus on Haiti (for other parts of the study also data from Mexico, Peru, USA are included)	CHWs	Multiple health focus<br><br>Promotion, prevention, curative	To describe the contribution of the non-governmental organization, Zanmi Lasante, to the HIV prevention and treatment scale-up and to the ongoing efforts to improve primary health care services in the public health system in Haiti	Mixed methods
Mutalemwa 2009 [109]	Tanzania	Community implementers, also known as Community Directed Distributors (CDDs)	Community Direction Intervention (CDI)<br><br>Multiple health focus<br><br>Distribution of drugs	To determine the extent to which the CDI process can be used for the delivery of other health interventions with different degrees of complexity	Qualitative
Nasreen 2011 [34]	Bangladesh	*Shasthya Kormi* (SK), *Shasthya Sebika* (SS), Newborn Health Workers (NHWs)	Maternal, neonatal and child health	To investigate whether a single dose of 400µg oral misoprostol could prevent PPH in a community home-birth setting and to assess its acceptability and feasibility among rural Bangladeshi women	Quantitative
Nelson 2012 [43]	South Sudan	Frontline Health Workers (FHWs), including Traditional Birth Attendants (TBAs), maternal-child-health workers, community midwives, CHWs	Mother and child health<br><br>Community based delivery assistance	To develop, implement, and evaluate an evidence-based Maternal, Newborn, and Child Survival package for FHWs in South Sudan	Mixed methods

Nsabagasani 2007 [77]	Uganda	Voluntary community based drug distributors	Malaria<br><br>Curative	To explore community perceptions, health worker and drug provider opinions of community based distribution of pre-packed antimalarials (HOMAPAK) and its effect on management of fever and use of other antimalarials	Qualitative
Nyanzi 2007 [32]	Gambia	Traditional Birth Attendants (TBAs)	Multiple<br><br>Prevention, promotion, curative	To understand the different roles that TBAs play in rural Gambia, exploring within and beyond metaphors of health in order to examine broader socio-cultural constructs	Qualitative
Olang'o 2010 [63]	Kenya	CHWs	HIV Home Based Care<br><br>Prevention, promotion, curative	To examine the attrition rates of CHWs from the HBC programme in Nyang'oma division, Bondo district, Nyanza province in western Kenya and to examine the trend, proximate and underlying causes and discuss the implications of attrition on the health care system and on support to those living with HIV and AIDS	Qualitative
Omer 2008 [92]	Pakistan	Lady Health Workers (LHWs)	Multiple	To demonstrate the effective use of community-based evidence for health promotion by LHWs in Sindh province, Pakistan	Quantitative
Osawa 2010 [64]	Zimbabwe	Care Facilitators (CFs)	HIV home based care<br><br>Prevention, promotion, curative	To understand the socio-demographic factors influencing the motivation and sustainability of CFs engaged in a community home-based HIV and AIDS programme, and the association between motivational outcomes, self-assessed performance, and CFs' perception toward the work and work environments in the community home-based HIV and AIDS programme in Masvingo Province, Zimbabwe	Quantitative
Peltzer 2010 [110]	South Africa	Lay HIV counselling and testing (HTC) counsellors	HIV<br><br>Prevention, promotion, counselling, testing	To evaluate the feasibility, fidelity, and effect of a HIV risk reduction intervention delivered to HIV-infected patients by lay counsellors during routine HCT public service in Mpumalanga, South Africa	Mixed methods
Perez 2009 [44]	Mali	CHWs	Child health<br><br>Promotion, preventive	To assess the performance of CHWs in the promotion of child health services at the household level in the district of Djenné, region of Mopti, Republic of Mali	Mixed methods

Posner 2009 [27]	Nepal	Peer Educators (PEs)	Caste-associated menstrual prohibitions and the vulnerability of adolescents girls and women to HIV<br><br>HIV risk awareness	To examine how self-efficacy and collective efficacy function to bring about individual and normative behavioural change among the adolescent girls who facilitated a non-formal education programme	Quantitative
Prata 2012 [37]	Bangladesh	Trained Traditional Birth Attendants (TBAs)	Maternal health, deliveries<br><br>Referral	To evaluate TBA's knowledge acquisition, knowledge retention and changes in attitudes and practices related to post-partum haemorrhage management in home births after undergoing training on the use of misoprostol and a delivery mat	Quantitative
Prata 2012b [35]	Nigeria	Community Oriented Resource Persons (CORPs), drug keepers, trained TBAs	Maternal health<br><br>Counselling, referral	To demonstrate the importance of community mobilization in the uptake of a health intervention, namely, community-based distribution of misoprostol to prevent post-partum haemorrhage	Quantitative
Puett 2013 [53]	Bangladesh	CHWs	Child health: immunization, acute respiratory infections, malnutrition<br><br>Prevention, promotion	To assess the quality of care provided by CHWs in managing cases of severe acute malnutrition by provision of community-based management of acute malnutrition protocols	Mixed methods
Rahman 2008b [65]	Bangladesh	*Shasthya Shebikas* (SS)	Multiple: maternal, neonatal and child health, including malaria, midwifery, tuberculosis, antenatal care<br><br>Prevention, promotion, curative	To explore whether and how the income earning capability varied among the new and old SSs, due to introduction of MNCH activities in the Nilphamari district of northern Bangladesh, including factors influencing their motivation and sustenance	Quantitative
Rahman 2010 [60]	Bangladesh	CHWs	Maternal and neonatal health<br><br>Prevention, promotion, curative	To assess factors affecting recruitment and retention of CHWs who were part of an intervention trial that evaluated effectiveness of two different service delivery models of a package of maternal and newborn care	Mixed methods

Razee 2012 [45]	Papua New Guinea	Various rural health workers: health extension officers, officers-in-charge, sisters-in-charge, CHWs and nursing officers	Not described, focus of article was on motivation and performance of health workers	To investigate how social factors impact on health worker motivation and performance in rural health services in Papua New Guinea	Qualitative
Root 2011 [54]	Swaziland	Trained caregivers	HIV and AIDS<br><br>Home care	To explore the concept of religious health assets and its relevance to HIV and AIDS	Qualitative
Sadler 2011 [55]	Bangladesh	CHWs	Child care<br><br>Prevention, curative	To examine the effectiveness and feasibility of adding diagnosis and treatment of Severe Acute Malnutrition to the Community Case Management package delivered by community health workers outside health facilities in Barisal, Bangladesh	Qualitative
Saleem 2007 [46]	Pakistan	Traditional Birth Attendants (TBAs)	Home deliveries	To determine the safety of 0.6% chlorhexidine vaginal and neonatal wipes and to estimate whether a randomized trial of 0.6% chlorhexidine vaginal and neonatal wipes could be conducted in home-delivery settings in Pakistan	Mixed methods (including a randomized controlled trial)
Sanghvi 2010 [36]	Afghanistan	CHWs	Maternal and neonatal health<br><br>Prevention of post-partum haemorrhage by distributing misoprostol	To test the safety, acceptability, feasibility, and effectiveness of community-based education and distribution of misoprostol by CHWs for prevention of postpartum haemorrhage at home birth in Afghanistan	Quantitative (non-randomized experimental design)
Sanjana 2009 [102]	Zambia	Lay counsellors	HIV<br><br>Counselling and testing	To review the effectiveness of lay counsellors in addressing staff shortages and the provision of HIV counselling and testing services	Mixed methods
Saravanan 2011 [28]	India	Trained Traditional Birth Attendants (TBAs)	Maternal health<br><br>Deliveries	To assess the ways in which a TBA training programme in India has been successful in disseminating evidence-based knowledge on birthing practices	Quantitative
Saravanan 2012 [47]	India	Traditional Birth Attendants (TBAs)	Design of a TBA training programme<br><br>Deliveries	To assess the extent to which there is a synthesis of both biomedical and locally practiced knowledge in the content and community involvement in the design of TBA a training programme in India.	Qualitative (literature review)

Satti 2012[1]	Lesotho	Traditional Birth Attendants (TBAs) (who became later clinic affiliated maternal health workers)	Maternal health<br><br>No info	To report the experience in rural Lesotho, where Partners in Health in partnership with the Lesotho Ministry of Health and Social Welfare has implemented a pilot programme that provides comprehensive care for pregnant women from the community to the health centre level, linking key primary care services (including HIV testing and treatment) to antenatal care and facility-based delivery	Quantitative, (uncontrolled before-after study)
Schneider 2008 [78]	South Africa	CHWs (as umbrella concept for amongst others Community Development Workers, Community Development Practitioners, Mid-level Worker, Community Caregivers, Child and Youth Care Workers, Youth Workers, Probation Officers/Community Service Officers and Early Childhood Development Practitioners)	Multiple health focus, including HIV, tuberculosis<br><br>Prevention, promotion	To examine the current generation of CHWs in South Africa in the light of the history and international experience with CHWs, with a focus on their central role in the response to HIV and AIDS, to analyse the national policy context and then report on the empirical reality of CHWs in the primary health care system of one of the nine provinces (Free State) of the country, and to discuss the effectiveness, tensions and prospects of sustainability of CHWs in the South African health system	Qualitative
Scott 2010 [29]	India	Accredited Social Health Activists (ASHAs)	Maternal and child health, family planning<br><br>Prevention, promotion, curative	To insights into how best to support CHW programmes	Qualitative
Shah 2010 [84]	Bangladesh	CHWs	Neonatal health<br><br>Prevention, cleaning of umbilical cord	To research practical implications and operational challenges associated with the deployment of large cadres of community-based workers within an efficacy trial of chlorhexidine for cleansing the umbilical cord	Qualitative (case study within a cluster randomized controlled trial)
Shankar 2009 [30]	Indonesia	Community facilitator	Maternal health (micronutrient deficiencies in pregnant women)<br><br>Promotion, preventive	To examine the additional health-care impacts that have resulted from the overall engagement of the Supplementation with Multiple Micronutrients Intervention Trial (SUMMIT) programme activities within the community and the role of the community facilitators in promoting positive health behaviours	Quantitative (randomized, double-blind, controlled clinical trial)

[1] Satti H, Motsamai S, Chetane P, Marumo L, Barry D, Riley J, McLaughlin M, Seung K, Mukherjee J: **Scaling up a comprehensive approach to improving maternal health in the mountains of lesotho.** *International Journal of Gynecology and Obstetrics* 2012, **119:**S472-S473.

Simba 2009 [66]	Tanzania	Peer educators	Reproductive health, Sexually Transmitted Infections<br><br>Education, support, referral	To explore the motive behind voluntarism among adolescent peer educators in Mbeya region, Tanzania with a view to making recommendations on strategies for sustaining peer education activities	Mixed methods
Simon 2009 [85]	Mozambique	*Agente Polivalente Elementar* (APE), tuberculosis volunteers, *Agente Comunitário de Saúde* (ACS), TBAs, HIV support groups	Multiple health topics<br><br>Prevention, support, curative	To present a participant-observer description of the evolution of community health worker support to the health services in Angonia district, Mozambique	Qualitative (descriptive)
Simwaka 2012 [112]	Malawi	Trained informal providers (shop owners)	Tuberculosis<br><br>Advise on medicine, referral	To determine the effectiveness and acceptability of a store keeper based referral system for tuberculosis suspects in urban settings of Lilongwe, Malawi	Mixed methods
Smith 2007 [93]	Pakistan	Different 'Support workers' (including Lady Health Workers (LHWs), Lady Health Visitors (LHVs), lady health assistants and CHWs)	Multiple (tuberculosis, maternal and child health)	To learn from Pakistan's experience with support workers to improve access of the UK system for ethnic minority groups	Qualitative
Smith 2013 [107]	Madagascar	Community Health Volunteers (CHVs)	Multiple<br><br>Prevention, promotion, curative	To synthesize the findings from a qualitative and a cross-sectional study on CHV programme functionality and performance in Madagascar	Mixed methods
Soofi 2012 [103]	Pakistan	Lady Health Workers (LHWs)	Single focus. Pneumonia in children aged 2-59 months<br><br>Prevention, treatment	To establish whether community case identification and management of severe pneumonia by oral antibiotics delivered through community health workers has the potential to reduce the number of infants dying at home	Quantitative (cluster randomized controlled trial)
Sranacharoenpong 2011 [94]	Thailand	Community Health Care Workers (CHCWs)	Diabetes and other diseases<br><br>Health promotion, basic health care	To investigate barriers to and support for implementing a community-based diabetes prevention education programme for CHCWs and to get preliminary input into programme design from the perspectives of health-care professionals and potential programme recipients of Chiang Mai province, Thailand	Qualitative

Srivastava 2009 [49]	Uttar Pradesh, India	Accredited Social Health Activists (ASHAs)	Antenatal care, postnatal care, maternal health<br><br>Primary medical care, education, counselling	To conduct a rapid appraisal of the functioning of ASHA in the community and her interface with community and service providers	Mixed methods
Suri 2007 [113]	South Africa	CHWs	Tuberculosis, HIV<br><br>Monitoring Directly Observed Treatment, Short-course, education, promotion	To examine the perspectives of CHWs to identify ways of improving the current CHW programme to more effectively combat the spread of HIV infection and tuberculosis in South Africa	Mixed methods
Takasugi 2012 [74]	Kenya	CHWs	Multiple health focus<br><br>Preventive, promotion	To examine determinants of work motivation of voluntary CHWs in Kenya	Qualitative
Teela 2009 [73]	Myanmar	Maternal Health Workers (MHWs)	Maternal Health<br><br>Prevention, promotion, curative	To evaluate the feasibility and impact of community-based provision of evidence-based maternal health interventions via the Mobile Obstetric Maternal Health Worker project in eastern Burma	Qualitative
Teklehaimanot 2007 [80]	Ethiopia	Health Extension Workers (HEWs)	16 packages of the Health Extension Package	To assess the working conditions of the first batch of HEWs (deployed in early 2005) and their job satisfaction	Qualitative
Uzochukwu 2008 [31]	Nigeria	Community Health Extension Workers, CHEWs)	Integrated Management of Childhood Illnesses (IMCI)	To assess if shorter training on IMCI will improve performance of health workers	Mixed methods
Viswanathan 2012 [56]	Afghanistan	CHWs	Family planning, antenatal care, maternal health<br><br>Prevention, promotion, curative	To determine if presence of a CHW in the community is associated with increased use of modern contraception, antenatal care and Skilled Birth Attendance in Afghanistan	Quantitative (household survey)

Winch 2008 [95]	Mali	CHWs	Integrated Management of Childhood Illnesses, Malaria<br><br>Prevention, promotion, curative	To: a) evaluate community promotion of zinc treatment and identify more effective channels of communication b) identify and resolve obstacles to implementation of zinc through community health centres and through a system of village drug kits managed by CHWs and c) identify factors that facilitate or impede the adoption of appropriate home management (treatment) of diarrhoea, including supplementation with zinc	Mixed methods
Wools-Kaloustian 2009 [71]	Kenya	Community Care Coordinators (CCCs)	HIV<br><br>Prevention, promotion, curative	To assess a model for extending antiretroviral care through CCCs, regarding acceptability and feasibility	Mixed methods
Ye-ebiyo 2007 [81]	Ethiopia	Health Extension Workers (HEWs)	Multiple	To make a clear needs assessment of continuing education and clearly map out and articulate priorities in and identify resources to undertake continuing education for HEWs	Qualitative
